# Cross-Linkable Polymer-Based Multi-layers for Protecting Electrochemical Glucose Biosensors against Uric Acid, Ascorbic Acid, and Biofouling Interferences

**DOI:** 10.1021/acssensors.3c00050

**Published:** 2023-03-21

**Authors:** Anna Lielpetere, Kavita Jayakumar, Dónal Leech, Wolfgang Schuhmann

**Affiliations:** † Analytical Chemistry−Center for Electrochemical Sciences, Faculty of Chemistry and Biochemistry, Ruhr University Bochum, Universitätsstr. 150, 44780 Bochum, Germany; ‡ School of Biological & Chemical Sciences, University of Galway, University Road, H91 TK33 Galway, Ireland

**Keywords:** glucose biosensor, electrochemical, zwitterionic polymer, biofouling, interferences

## Abstract

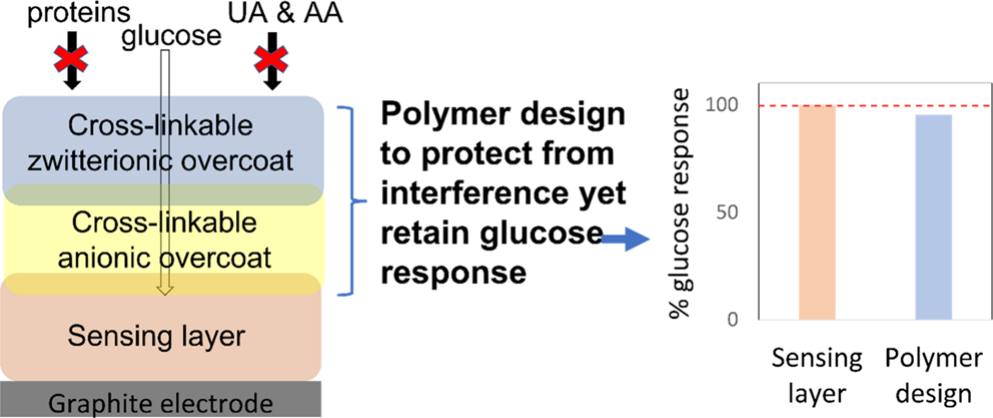

The lifetime of implantable electrochemical glucose monitoring devices is limited due to the foreign body response and detrimental effects from ascorbic acid (AA) and uric acid (UA) interferents that are components of physiological media. Polymer coatings can be used to shield biosensors from these interferences and prolong their functional lifetime. This work explored several approaches to protect redox polymer-based glucose biosensors against such interferences by designing six targeted multi-layer sensor architectures. Biological interferents, like cells and proteins, and UA and AA interferents were found to have individual effects on the current density and operational stability of glucose biosensors, requiring individual protection and treatment. Protection against biofouling can be achieved using a poly(2-methacryloyloxyethyl phosphorylcholine-*co*-glycidyl methacrylate) (MPC) zwitterionic polymer coating. An enzyme-scavenging approach was compared to electrostatic repulsion by negatively charged polymers for protection against AA and UA interferences. A multi-layer novel polymer design (PD) system consisting of a cross-linkable negatively charged polyvinylimidazole-polysulfostyrene co-polymer inner layer and a cross-linkable MPC zwitterionic polymer outer layer showed the best protection against AA, UA, and biological interferences. The sensor protected using the novel PD shield displayed the lowest mean absolute relative difference between the glucose reading without the interferent and the reading value with the interferent present and also displayed the lowest variability in sensor readings in complex media. For sensor measurements in artificial plasma, the novel PD extends the linear range (*R*^2^ = 0.99) of the sensor from 0–10 mM for the control to 0–20 mM, shows a smaller decrease in sensitivity, and retains high current densities. The application of PD multi-target coating improves sensor performance in complex media and shows promise for use in sensors operating in real conditions.

## Introduction

Due to the prevalence of diabetes in society, there has been an increasing need for devices that monitor glucose levels in blood.^1^ Research efforts and advances in technology over the past decade have seen the progression of these monitoring devices from fledgling fingerprick devices, relying on test strips inserted into a detector,^2,3^ to subcutaneous or fully implantable continuous glucose monitors (CGMs).^4,5^ Of the commercially available CGMs, those based on electrochemical biosensing dominate the market due to their specificity, fast response time, sensitivity, and low detection limit.^6,7^ While they provide an alternative to daily fingerprick tests, CGMs present challenges, mostly originating from the hostile environment on implantation and the complexity of physiological fluids. Foreign body response (FBR) is the host’s immune reaction to an implanted device that results in inflammation and, ultimately, encapsulation of the device by a fibrotic capsule, which negatively affects the accuracy, sensitivity, and lifetime of in vivo glucose biosensors.^8^ Electrochemical biosensors, in particular, suffer losses in sensitivity, accuracy, and sensor drift as sensor operation relies upon the diffusion of the analyte to-and-from the sensor surface—a process affected by the progressive growth of the fibrotic capsule over time.^9^ Hindered diffusion of the product due to the fibrotic capsule can lead to the accumulation of gluconic acid, resulting in a drop in the local pH, influencing the enzyme’s stability and thereby the sensor’s lifetime. Additionally, the biological cells consume glucose, leading to a local decrease in the glucose concentration, thereby affecting the sensor response. The lifetime of currently available electrochemical CGMs does not exceed 14 days after implantation.^10^ Moreover, inflammation leads to the degradation of implant components and further reduces their lifetime.^11^

New materials are being developed to reduce the effects of FBR and prolong the functional lifetime of biosensors.^8,10,12^ A promising class of materials are zwitterionic polymers that reduce non-specific protein adsorption by impairing electrostatic interactions and forming a hydration layer.^13^ We recently reported that a 2-methacryloyloxyethyl phosphorylcholine-based zwitterionic polymer (MPC) coating containing cross-linkable epoxy functional groups decreases fibrinogen adsorption and fibroblast adhesion in *in vitro* tests, while improving the performance and operational stability of glucose biosensors when used as a protective coating.^14^

Endogenous and exogenous small-molecule interferences can also affect the sensor signal.^6,15^ Low-molecular-weight species like ascorbic acid (AA) and uric acid (UA) can be directly oxidized at the electrode and/or inhibit the glucose-oxidizing enzyme, affecting its bioelectrocatalytic activity.^16^ Uric acid has also been reported to act as an uncompetitive inhibitor of glucose-converting enzymes such as glucose oxidase (GO*x*), FAD-dependent glucose dehydrogenase (FAD-GDH), and cellobiose dehydrogenase (CDH).^17^ Several strategies have been developed for improving the sensitivity and performance of these glucose biosensors, including coating with (i) permselective membranes that discriminate against interfering species either by size or charge, (ii) layers to remove oxidizable species, as well as (iii) coatings that release anti-inflammatory agents to attempt to prevent FBR.^15^

The majority of these approaches rely on integration of a polymeric shield into a sensor architecture. Protective membranes have been used on glucose biosensors since the first commercial glucose biosensor employed an inner cellulose acetate and an outer polycarbonate membrane.^3^ Anionic membranes such as Nafion or poly(ester-sulfonic acid)^18,19^ that repel the negatively charged AA and UA interferents are often used. Multi-layer membranes consisting of Nafion and cellulose acetate or polyurethane can protect against a wider range of interferents.^18,20^ These membranes are mainly useful to protect first-generation glucose biosensors that detect hydrogen peroxide due to the low permeability of the multi-layers to larger molecules such as the glucose target analyte. The application of coatings is detrimental to electrochemical biosensor response, as they lead to the formation of a diffusional barrier.^21−23^ A major challenge that remains is to improve the selectivity of electrochemical glucose biosensors while maintaining high sensitivity, stability, and response time.^24^

Integration of additional enzyme scavenging layers in the sensing strategy to remove oxidizable species has been attempted. For example, a layer using a combination of glucose oxidase and catalase has been successful in removing glucose as an interferent in electrochemical enzymatic biosensors detecting another analyte, such as sucrose or lactose.^25,26^ In addition, a horseradish peroxidase (HRP) layer can diminish interference from AA, UA, and acetaminophen in glucose sensors by oxidizing them in the presence of hydrogen peroxide.^27,28^ A layer of HRP in combination with lactate oxidase (LO*x*) has been used in the commercial FreeStyle Navigator sensor to generate peroxide in situ for the oxidation of these interferents, although a drawback of this method is the reliance on dissolved oxygen as the LO*x* co-substrate.^3^ Ascorbate oxidase (AsO*x*) can also selectively remove AA in the presence of an oxygen co-substrate and has been employed for sample treatment.^29−31^ Interference from AA has also been minimized by the incorporation of bovine serum albumin (BSA) or phospholipids in a poly(*o*-phenylenediamine)-containing GO*x* layer on platinum used for in vivo brain monitoring of glucose. The AA interferent was blocked by the BSA or phospholipid because they increased the density of the film without increasing the demand for oxygen.^32^ Enzyme integration for use as a scavenging system has a limitation: there is a possibility of wiring AsO*x* to the electrode either via mediator or direct electron transfer. The AsO*x* layer must therefore be separated from the mediating sensing layers and/or electrodes, usually achieved by application of a redox-silent polymer between the sensing and the enzyme-scavenging layer. This, however, leads to the formation of a higher diffusional barrier for glucose. Moreover, the efficiency and duration of the interferent-removal layer are limited by enzyme activity and lifetime.

The protective measures are not multi-purpose, i.e., a protective coating may target minimizing either biological interference or interference from low-molecular-weight species, but not both. Multi-purpose protection can be achieved either using a single layer system with a multi-purpose effect—minimizing biological and low-molecular-weight species interference—or a multi-layer sensor architecture, wherein each consecutive layer has its own role in minimizing a specific effect. Regardless of which of these architectures is successful, the protective measure should minimize loss in the sensor signal.

Here, we report on approaches to protect redox polymer-based electrochemical glucose biosensors against biological interference, using BSA as a model globular protein interferent, and low-molecular-weight AA and UA interferences by designing targeted multi-layer, cross-linkable, sensor architectures. Protection against biofouling is realized by using a previously designed zwitterionic polymer incorporating epoxy cross-linking functional groups.^14^ Two different strategies are examined for the removal of UA and AA interferences: an enzymatic scavenging approach and a negative polymer layer approach.

## Materials and Methods

### Chemicals

All chemicals were purchased from Sigma-Aldrich, Alfa Aesar, Acros Organics, TCI, or Roth and used as received unless otherwise noted. Glycidyl methacrylate and butyl acrylate were passed through a column containing the corresponding inhibitor remover (Sigma-Aldrich) and stored at −20 °C prior to use. The polymerization initiator azobisisobutyronitrile (AIBN) was recrystallized from hot toluene and stored at −20 °C prior to use. The redox polymer [poly(1-vinylimidazole)Os(bpy)_2_Cl]^+^ (Os(bpy)PVI) was synthesized by the modification of published procedures.^33,34^ A cellobiose dehydrogenase (CDH) from *Crassicarpon hotsonii* (syn. *Myriococcum thermophilum*) modified with glucose activity-enhancing mutations C291Y and W295R provided by DirectSens GmbH^35^ was used as the glucose oxidizing enzyme in this study. Uricase from *Bacillus fastidiosus* (specific activity ∼9 U/mg) and AsO*x* from *Cucurbita* species (specific activity ≥1500 U/mg) were purchased from Sigma-Aldrich. All aqueous solutions were prepared using water purified and deionized with a Milli-Q system.

**Scheme 1:**
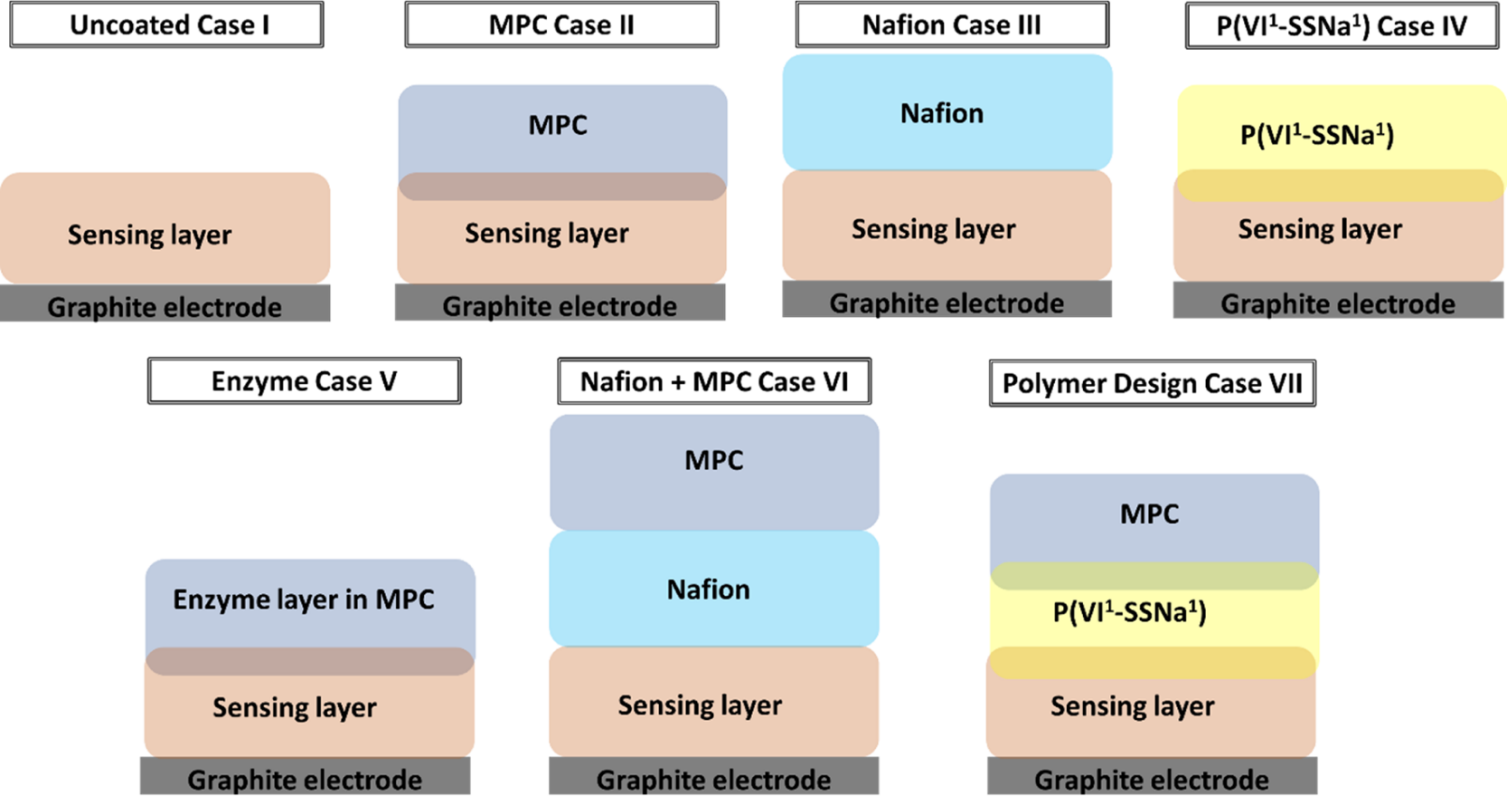
Schematic of Different Sensor Architectures

### Synthesis

All reactions and manipulations were conducted using a standard Schlenk technique under an argon atmosphere. The co-polymer structures and nomenclature are presented in Figure S1. Polymers were characterized by NMR, with results provided in Figures S2–S4. The synthesis of the P(SS-GMA-BA) was described previously.^26^ The synthesis of the zwitterionic 2-methacryloyloxyethyl phosphorylcholine (MPC)-based co-polymer containing cross-linkable epoxy functional groups in the co-polymer was as described previously.^14^

#### Poly(1-vinylimidazole-*co*-4-styrene sulfonic acid sodium salt hydrate) P(VI^12^-SSNa^1^)

The synthesis procedure has been published previously.^36^ Briefly, 1-vinylimidazole (3.31 mL, 37 mmol) and 4-styrene sulfonic acid sodium salt hydrate (0.62 g, 3 mmol) were dissolved in DMSO/H_2_O 1:1 (4 mL) in a round-bottom flask equipped with a condenser and deaerated by argon bubbling; then, AIBN (0.25 g, 1.5 mmol) was added. The reaction mixture was heated to 70 °C. After 30 min, a yellow gel was obtained and heating was stopped. After cooling to room temperature, the crude product was dissolved in 20 mL of EtOH and 3 mL of H_2_O. The solution was poured into stirred acetone (80 mL), and the white precipitate was separated by centrifugation and dried under reduced pressure for 24 h. Off-white solid (3.11 g). ^1^H NMR (200.13 MHz, D_2_O) δ/ppm: 6.71–7.69 (overlapping signals, broad, imidazole, benzene), 3.88 (s, broad, CH–N), 3.23 (s, broad, CH), 2.12 (s, broad, CH_2_, CH_3_).

#### Poly(1-vinylimidazole-*co*-4-styrene sulfonic acid sodium salt hydrate) P(VI^1^-SSNa^1^)

As above, 1-vinylimidazole (453 μL, 5 mmol) and 4-styrene sulfonic acid sodium salt hydrate (1.03 g, 5 mmol) were dissolved in DMSO/H_2_O 1:1 (10 mL) in a round-bottom flask equipped with a condenser and deaerated by argon bubbling; then, AIBN (0.25 g, 1.5 mmol) was added. The reaction mixture was heated to 70 °C for 1 h. After cooling down, the product was precipitated by pouring into stirred acetone (∼150 mL), separated by centrifugation, washed with acetone, and freeze-dried under high vacuum. White solid (1.24 g). ^1^H NMR (300 MHz, D_2_O) δ/ppm: 7.89–7.21 (m, 2H), 7.05–6.21 (m, 2H), 2.15–0.98 (m, 3H).

### Electrode Modification

Graphite rods (Graphite store, USA, 4.0 mm diameter, NC001300) were cut, insulated with heat shrink tubing, and polished at one end using fine grit paper to give graphite disk working electrodes with a geometric surface area of 0.126 cm^2^. The uncoated control Case I biosensors were assembled, as described previously.^14^ The deposition was allowed to dry for 3 h before a 10 μL aliquot of 0.5 wt./v % of the selected polymer coating was applied to the electrodes. The electrodes were allowed to cure overnight at ambient temperature before being used. For multi-layer systems, an additional coating of 10 μL aliquot of 0.5 wt./v % of the selected additional polymer was applied 1 h after addition of the first polymer coating. For the enzyme layer, uricase (15 μg, 9 U/mg) and AsO*x* (15 μg, ≥1500 U/mg) were mixed with 8 μL of MPC aliquot. First, 2 μL of MPC was added as a coating and allowed to dry before addition of the enzyme/MPC mixture.

### Electrochemical Measurements

Electrochemical tests were conducted using a CH Instrument 1030a multichannel potentiostat (IJ Cambria) with enzyme electrodes as working electrodes, a custom-built Ag|AgCl (3 M KCl) reference electrode, and a platinum mesh (Goodfellow) counter electrode placed in an electrochemical cell thermostated at 37 °C; experiments were conducted, unless otherwise stated, in the presence of ambient oxygen. Current signals are normalized to the geometric surface area of the graphite disk electrodes to generate current density data.

Artificial plasma was prepared based on an aqueous solution recipe of uric acid (68.5 mg L^–1^), ascorbic acid (9.5 mg L^–1^), fructose (36 mg L^–1^), lactose (5.5 mg L^–1^), urea (267 mg L^–1^), cysteine (18 mg L^–1^), sodium chloride (6.75 g L^–1^), sodium bicarbonate (2.138 g L^–1^), calcium sulfate (23.8 mg L^–1^), magnesium sulfate (104.5 mg L^–1^), and bovine serum albumin (7 g L^–1^).^37^

Interference screening data was analyzed by calculating the mean absolute relative difference (MARD) between the mean baseline glucose concentration reading without the interferent (*M*_0_) and the mean glucose reading value with the interferent present (*M*_I_) as
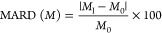
1

In this study, interference was defined as an absolute MARD ≥20%.^38^ Testing levels for UA, BSA, and AA were set at their concentrations in artificial plasma. The effect of UA and AA on the biosensor signal was tested using amperometry at 0.35 V in 5 mM glucose with injections of UA or AA into the electrochemical cell. The effect of BSA on the biosensor signal was tested using amperometry in 5 mM glucose prior to and then 10 min after adding BSA into the electrochemical cell.

## Results and Discussion

We previously reported that a zwitterionic 2-methacryloyloxyethyl phosphorylcholine (MPC)-based co-polymer, containing cross-linkable epoxy functional groups in the co-polymer, is the most effective coating over a glucose biosensor to prevent fibrinogen adsorption and biological cell adsorption while maintaining good sensor performance and improved sensitivity in buffer solution and in the presence of BSA.^14^ Here, we explore the design of sensor architectures to control not only the adsorption of biological molecules but also to limit UA and AA interference to the sensor signal while preserving efficient substrate and product mass transport to maintain a high sensor signal.

### Design of Protective Systems

Three characteristics were considered in the design of glucose sensors to protect against interferents present in physiological media: (i) resistance to biofouling; (ii) resistance to interference from AA and UA; and (iii) efficient mass transport of substrate and product through the layers. A total of seven cases providing protection against interference were examined, as presented in Table 1 and Scheme 1.

**Table 1:** Predicted Performance of Glucose Biosensor Systems for Each Sensor Architecture

Case	protection against biological interference	protection against UA and AA interference	efficient glucose and gluconic acid transport
I (uncoated)	–	–	+
II (MPC)	+	–	+
III (Nafion)	–	+	–
IV (P(VI^1^-SSNa^1^))	–	+	+
V (enzyme layer)	+	+	+
VI (Nafion + MPC)	+	+	–
VII (polymer design)	+	+	+

A glucose biosensor consisting of an enzyme embedded in a cross-linked redox polymer hydrogel (Case I) is susceptible to non-specific protein adsorption and cell adhesion when operating in physiological fluids. This occurs within minutes to hours, and the adsorbed and adhered biological film creates a diffusional barrier to glucose, leading to sensor signal drift consistent with the changes in glucose diffusivity.^39^ Additionally, species such as AA and UA can diffuse through the sensing layer to be oxidized at the electrode causing sensor interference.

A protective overcoat consisting of the zwitterionic MPC polymer (Case II) limits non-specific protein adsorption and cell adhesion while presenting a low diffusional barrier to glucose and charge transport. This is due to inter-layer mixing between the MPC and the sensing layer, driven by epoxy cross-linking.^14^ However, zwitterionic polymers may not hinder the diffusion of AA and UA as the swellability and high ionic conductivity of zwitterionic hydrogels enable rapid diffusion.^40^

Protection against negatively charged UA and AA interferences can be realized by electrostatic repulsion provided by a negatively charged polymer layer (Case III and Case IV). To test for this, a selection of polymers was chosen that included Nafion (Case III) and three polymers containing styrene sulfonate groups in various loadings and chemical compositions: a poly(styrene sulfonate-*co*-glycidyl methacrylate-*co*-butylacrylate) P(SS-GMA-BA) and two poly(1-vinylimidazole-*co*-styrene sulfonate) polymers P(VI^12^-SSNa^1^) and P(VI^1^-SSNa^1^) (see Supporting Information, Figure S1), where the superscripts indicate the monomer molar ratios. The P(SS-GMA-BA) polymer was previously used as a capping layer for improving the stability of bioelectrodes.^41^ High loading of sulfonate groups ensures good solubility in water to aid film deposition and swelling of the coating in aqueous solution to permit substrate and product diffusion,^42,43^ while the presence of epoxy cross-linking sites and hydrophobic methacrylate groups can be used to form a stable coating on surfaces. A (P(VI^12^-SSNa^1^)) was prepared to provide a polymer with a high loading of 1-vinylimidazole groups and therefore structural similarity to the Os(bpy)PVI redox polymer. Due to its nucleophilicity, 1-vinylimidazole can react with the PEGDGE cross-linker used to cross-link the redox polymer and enzyme to covalently anchor the poly(1-vinylimidazole-*co*-styrene sulfonate) film to the sensing layer. This may minimize the diffusional barrier introduced by the additional polymer layer.^14^ The P(VI^1^-SSNa^1^) had a lower loading of 1-vinylimidazole groups and a higher loading of the negatively charged styrene sulfonate groups. Amperometric tests with injections of the UA and AA interferents show the P(VI^1^-SSNa^1^)-coated sensor to be the most selective for glucose (Figure S5), and it was therefore chosen as the negatively charged polymer for further tests (Case IV). While polymer coatings of negatively charged polymers such as Nafion and P(VI^1^-SSNa^1^) can protect against interference from negatively charged substances such as AA and UA, they do not protect against protein adsorption or cell adhesion.^14^ Coating with polymers such as Nafion has also been shown to limit glucose diffusion.^14,20−23,44^

An enzymatic scavenging approach based on AsO*x* and uricase (UO*x*) (Case V) cross-linked in the MPC layer was also tested to protect from AA and UA interference by converting AA to 2-dehydroascorbate (by AsO*x*) and UA to 5-hydroxyisourate (by UO*x*) which further decomposes into allantoin. It is proposed that the MPC minimizes biofouling while retaining efficient glucose transport and that the enzymes scavenge UA and AA, providing protection from these interferents. This represents multi-purpose protection by a single layer.

An alternative multi-purpose protection using a multi-layer approach, integrating a negatively charged polymer layer between the sensing layer and the zwitterionic MPC layer, was tested to protect against AA and UA as well as protein and cell adhesion interferences. A multi-layer consisting of MPC and either Nafion (Case VI) or the P(VI^1^-SSNa^1^) polymer (Case VII) as the negatively charged polymer layer was selected for testing. As Nafion layers are known to limit glucose diffusion, it is predicted that the Nafion + MPC-coated Case VI sensor provides protection from interference but delivers a lower glucose sensor response. The novel polymer design in Case VII, using layers that can be cross-linked to each other, is predicted to protect from interference but delivers efficient mass transport of glucose to retain a strong glucose sensor response.

### Interference Protection Using a Zwitterionic Polymer or a Charged Polymer

Biosensors coated with the protective layers were investigated in PBS, artificial plasma, and PBS containing UA or BSA. BSA is known to adsorb non-specifically on sensors, mimicking the effect of human serum albumin (HSA) on biosensor current, and UA is known to affect the current response of enzymatic biosensors.^44^ All coated biosensors were compared to the non-coated electrode, Case I, that contained only the sensing layer of Os(bpy)PVI, CDH, and PEDGDE as an uncoated “control”.

**Figure 1:**
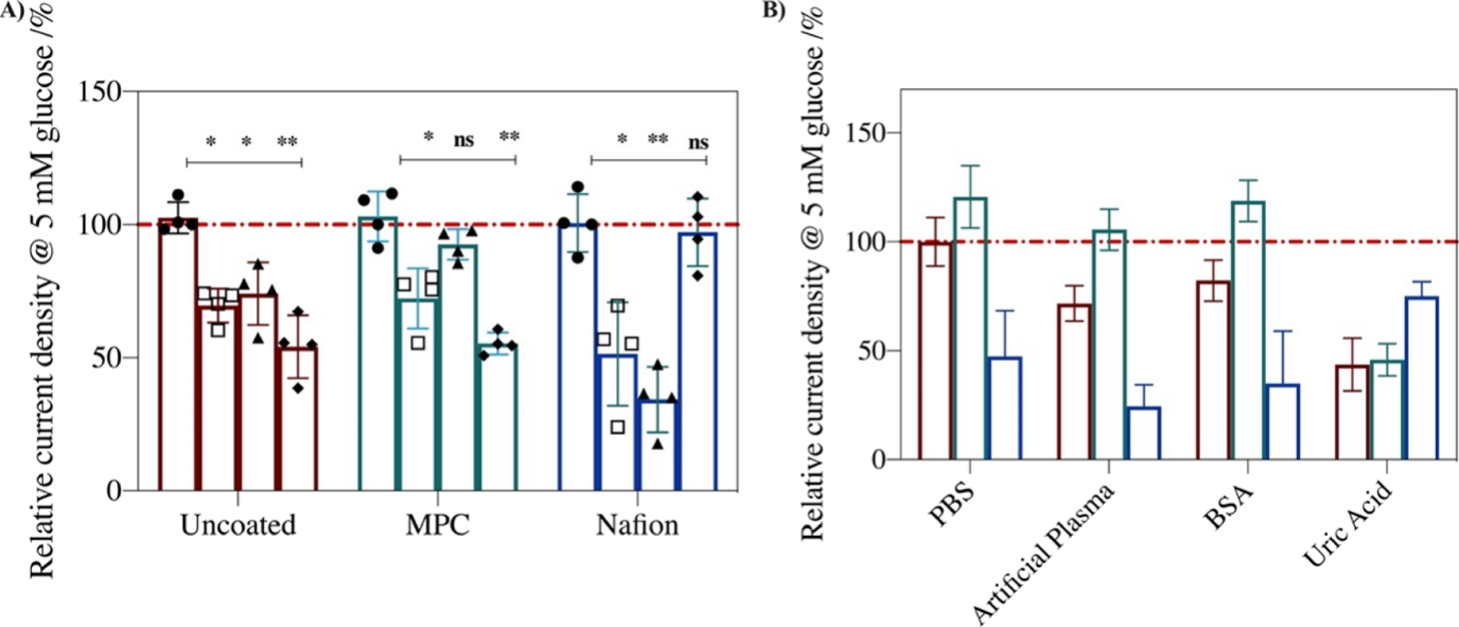
Relative current density for the detection of 5 mM glucose at 37 °C where (A) the current density in PBS (●), artificial plasma (□), BSA (▲), and UA (◆) is normalized to that of each system in PBS. (B) Current density for the detection of 5 mM glucose is normalized to the uncoated Case I control response in PBS. Responses are for uncoated control (Case I, maroon), MPC-coated (Case II, green), and Nafion-coated (Case III, dark blue) systems. Mean ± SD (*n* = 4); *p < 0.03, **p < 0.002, ***p < 0.0002.

Current densities for glucose oxidation at sensors coated with polymer coatings in PBS, artificial plasma, and PBS with BSA or UA relative to the response of the sensor in PBS (A) or to the response of the uncoated control (Case I) in PBS (B) are presented in Figure 1. The uncoated control (Case I) displays a significant drop in current density in UA (50%) and a 25 and 20% loss in artificial plasma and BSA, respectively. The current density decrease in UA is greater than that in artificial plasma with the same UA concentration. This may be because protein adsorption in artificial plasma hinders the diffusion of UA to the sensing layer. The same trend of current density decrease was previously observed for redox polymer-based enzyme electrodes prepared using either FAD-GDH or CDH as enzymes.^17^ The current decrease in the presence of UA may originate from the accumulation of the UA oxidation product allantoin produced by direct oxidation of UA at the electrode or from inhibition of the enzyme by UA. The higher baseline current in the absence of glucose than when UA is present (Figure S6) implies that the oxidation of UA also occurs.

Upon application of a protective polymer coating, the current density usually decreases due to the formation of a diffusional barrier, as reported previously.^14,44^ This is observed (Table S1) when Nafion (Case III) is used as a coating. The MPC coating (Case II) results, however, in an increase in current density relative to the response of the uncoated control Case I sensor (Figure 1B) and a decrease in *K*_m_^app^ (Table S1). This is likely due to the high ionic conductivity of the zwitterionic polymers and the interlayer mixing with the sensing layer because of epoxy cross-linking.^14^ In Case II, the sensor retains glucose oxidation current density in BSA but shows a significant sensor signal decrease in UA (45%) and artificial plasma (28%). This confirms that while the zwitterionic functionality of MPC enables protection from protein adsorption, the swellable nature does not inhibit the diffusion of and interference from low molecular-weight species such as UA.

For the Nafion coating, Case III, there is a statistically insignificant change in glucose oxidation current density in UA (−3%) but a significant decrease in artificial plasma (49%) and in BSA (65%). Nafion has a mixed hydrophilic/hydrophobic structure and is unable to form a strong hydration sphere to repel protein adsorption.^45,46^ Moreover, it is anionic and can electrostatically interact with positive domains of proteins, increasing protein adsorption. Thus, higher protein adsorption occurs,^14^ forming an additional layer which hinders the diffusion of the glucose substrate. The glucose *K*_m_^app^ and *j*_max_ remain the same in UA, indicating that Nafion coating hinders UA diffusion to the extent that the UA concentration in the sensing layer is insufficient to cause enzyme inhibition.

Coatings targeting the minimization of biofouling operate via the formation of a strong hydration sphere that forms an entropic barrier that proteins and cells must overcome for adsorption to occur,^40^ whereas protective coatings against UA and AA interference rely on electrostatic repulsion.^15^ An efficient system for protection from UA, AA, and protein adsorption in complex media will need to confer the properties of both Case II and Case III to be successful.

**Figure 2:**
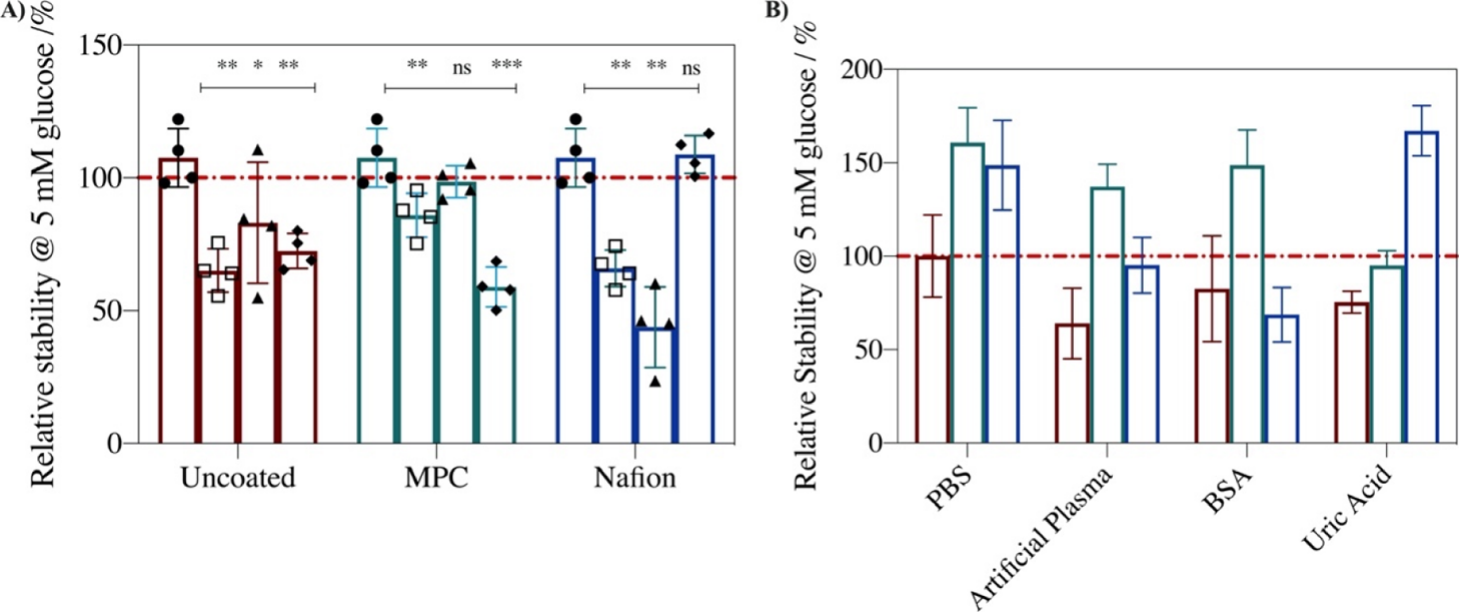
Relative stability after 12 h continuous polarization in 5 mM glucose, where (A) stability for PBS (●), artificial plasma (□), BSA (▲), and uric acid (◆) is normalized to that of each system in PBS (0.05 M, pH 7.4). (B) Stability is normalized to the control in PBS at 37 °C for uncoated control (Case I, maroon), MPC-coated (Case II, green), and Nafion-coated (Case III, dark blue) systems. Mean ± SD. (*n* = 4); *p < 0.03, **p < 0.002, ***p < 0.0002.

Considering the operational stability of the amperometric response to glucose oxidation, each system presents a lower sensor operational stability in complex media relative to that in PBS alone (Figure 2A). For example, the uncoated Case I shows a stability decrease of 35, 17, and 28% in artificial plasma, BSA, and UA, respectively. The MPC Case II retains sensor signal in BSA but displays significant decreases of 40% in UA and 15% in artificial plasma. However, the Nafion Case III shows a statistically insignificant decrease in operational stability in UA but a significant decrease of 57% in BSA and 35% in artificial plasma. For the MPC Case II and Nafion Case III, the presence of the individual polymer coating improves the operational stability of the amperometric response to glucose oxidation compared to that of the uncoated control Case I in PBS (Figure 2B).

### Multi-purpose Protection from Protein, UA, and AA Interferents

**Figure 3:**
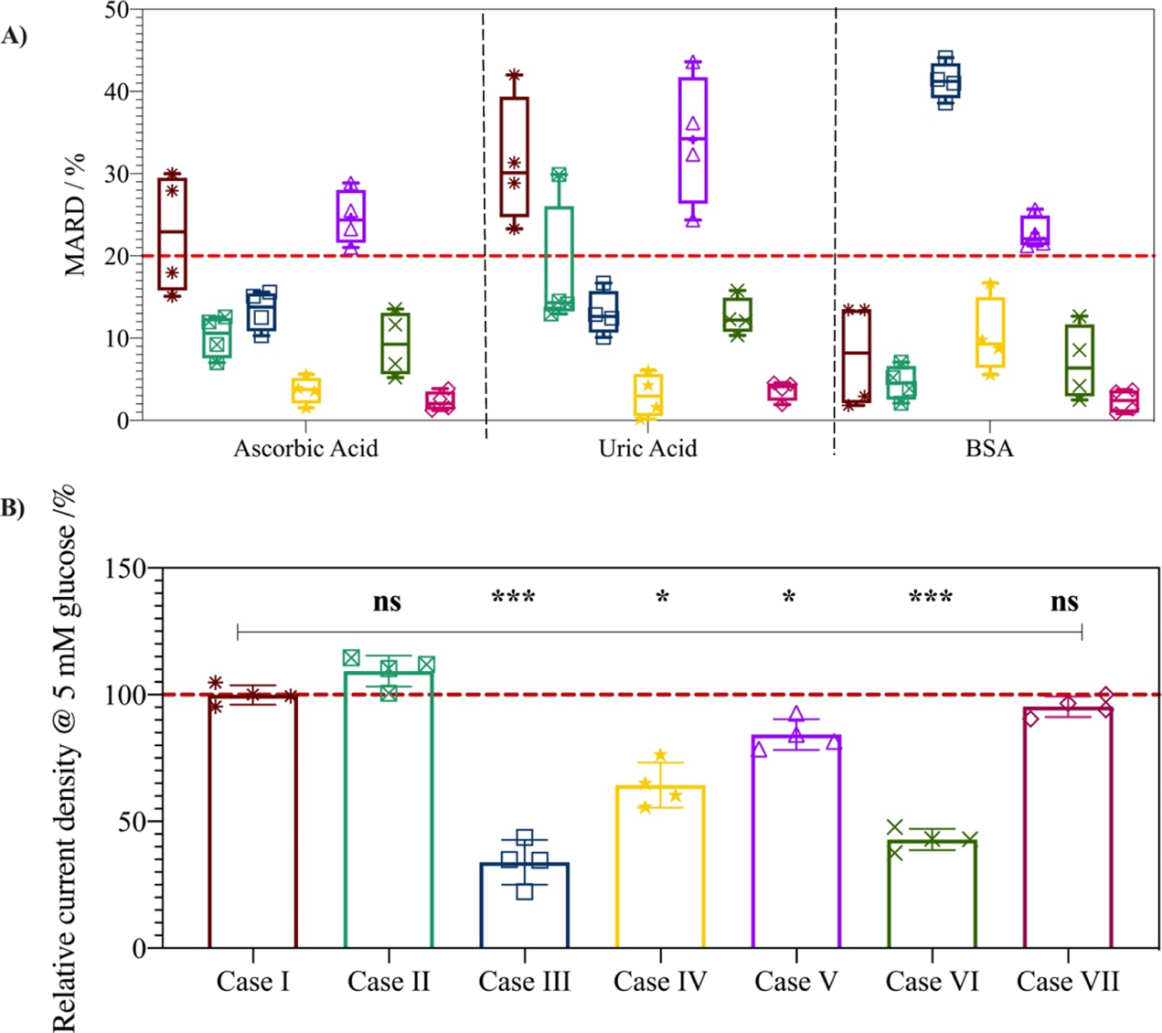
(A) Box plot showing the mean absolute relative difference (MARD, %) of the current signal in 5 mM glucose (PBS, pH 7.4, 37 °C) in the presence of ascorbic acid, uric acid, and BSA for uncoated control Case I (maroon, *), MPC-coated Case II (green, 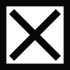), Nafion-coated Case III (dark blue,□), P(VI^1^-SSNa^1^)-coated Case IV (yellow,★), enzyme layer Case V (violet, △), Nafion + MPC-coated Case VI (dark green, ×), and novel polymer design Case VII (pink, ◇). The line inside the box = mean, box limits = standard deviation (*n* = 4); * and the symbols represent individual data points; lower and upper error bars = 5 and 95% limits, respectively; red line = 20% MARD threshold for the definition of interference. B) % current densities of Case I–VII in 5 mM glucose in PBS relative to that of the response of the uncoated control Case I in 5 mM glucose in PBS. Mean ± SD. (*n* = 4); *p < 0.03, **p < 0.002, ***p < 0.0002.

An interference screening was performed to evaluate if multi-purpose Case IV–VII protective coatings can minimize protein, AA, and UA interferences compared to the Case I–III protective coatings. Interference is calculated using a mean absolute relative difference (MARD) threshold of 20% in the presence of 5 mM glucose (eq 1). The MARD was selected as a measure as it quantifies the mean absolute difference in the presence of interference to the mean sensor response in the absence of interference. Additionally, the size of the box in Figure 3A represents the standard deviation of the sensor response of four electrodes. Sensor variability, such as a high standard deviation, can affect the sensitivity of the sensor or the ability to distinguish between successive glucose concentrations. The desired response is therefore a Case system that does not breach the 20% MARD threshold and displays a compact box indicating low variability in sensor readings across a sample size of 4.

The AA and UA, as expected, act as interferents at the uncoated control (Case I) with MARD of 23 ± 7 and 31 ± 8%, respectively. While protein adsorption may occur in BSA, the effect on the Case I sensor current is insufficient for BSA to be classified as an interferent (MARD = 8 ± 6%). However, the standard deviation is high for all Case I sensors. Moreover, as protein adsorption can increase with time, further protein adsorption may occur should a uncoated control Case I sensor be used over longer periods in complex media. For the MPC Case II system, UA acts as an interferent (MARD = 18 ± 8%) but AA and BSA have MARDs of 10 ± 2% and 5 ± 2%, respectively, and are not classified as interferents. The Nafion Case III system protects against interference from UA and AA (MARD = 13 ± 2%) but shows interference from BSA (MARD = 41 ± 2%). This can again be attributed to the mixed hydrophobic/hydrophilic structure and the anionic nature of Nafion.^47^

The novel synthesized P(VI^1^-SSNa^1^) co-polymer used in Case IV provides protection from UA, AA, and BSA interference with MARDs of 4 ± 2, 3 ± 2, and 10 ± 5%, respectively. While protein adsorption does occur, similar to the uncoated control Case I, the effect on the current is insufficient for BSA to be classified as an interferent. The difference in the effect of protein adsorption for P(VI^1^-SSNa^1^) Case IV versus Nafion Case III is likely due to the more hydrophilic nature of P(VI^1^-SSNa^1^) compared to Nafion, as hydrophobic films show greater biofouling than hydrophilic films.^45,48^ Nevertheless, as in the uncoated control Case I, the P(VI^1^-SSNa^1^) Case IV system response in BSA shows a relatively high standard deviation.

The enzyme-scavenging Case V approach results in interference from UA, AA, and BSA with MARDs of 25 ± 3, 34 ± 8, and 22 ± 7%, respectively. The interference from UA is likely due to low enzymatic activity of UO*x* (9 U/mg), which limits the efficiency of the enzymatic scavenging of UA. While the AsO*x* enzyme has good specific activity (1500 U/mg), amperometric tests (Figure S7) confirm that the ASO*x* system does not sufficiently protect against interference from AA, as the injection of AA leads to an increase in sensor current. It is interesting to note that MARD in the presence of BSA is higher for enzyme-scavenging Case V, where the enzymes are in the MPC layer, compared to MPC Case II of a layer only of MPC. It is possible that the presence of enzymes affects the surface chemistry sufficiently to diminish the protection against protein adsorption offered by a zwitterionic polymer.

The multi-layer architectures (Nafion + MPC Case VI and the novel polymer design Case VII) both show good protection from interference by UA, AA, and BSA with MARDs of 9 ± 4, 12 ± 2, and 7 ± 5% for Nafion + MPC Case VI and 2 ± 1, 3 ± 1, and 2 ± 1% for novel polymer design Case VII, respectively. Thus, from the results in Figure 3A, P(VI^1^-SSNa^1^) Case IV, Nafion + MPC Case VI, and the novel polymer design Case VII show the most promise as polymer shields for protection from interference from AA, UA, and biological interferences such as BSA. However, for P(VI^1^-SSNa^1^) Case IV and Nafion + MPC Case VI, the glucose oxidation current density is significantly lower than that observed at the non-coated control Case I (36 and 58% decrease, respectively) compared to that for the novel polymer design Case VII (Figure 3B). In addition, the novel polymer design Case VII system displays the lowest MARD and the lowest standard deviation of sensor signal. Therefore, the novel polymer design Case VII shows the best ability to protect against AA, UA, and BSA interferences, displays the lowest signal variability, and retains the highest sensor signal.

### Sensor Performance and Operational Stability

**Figure 4:**
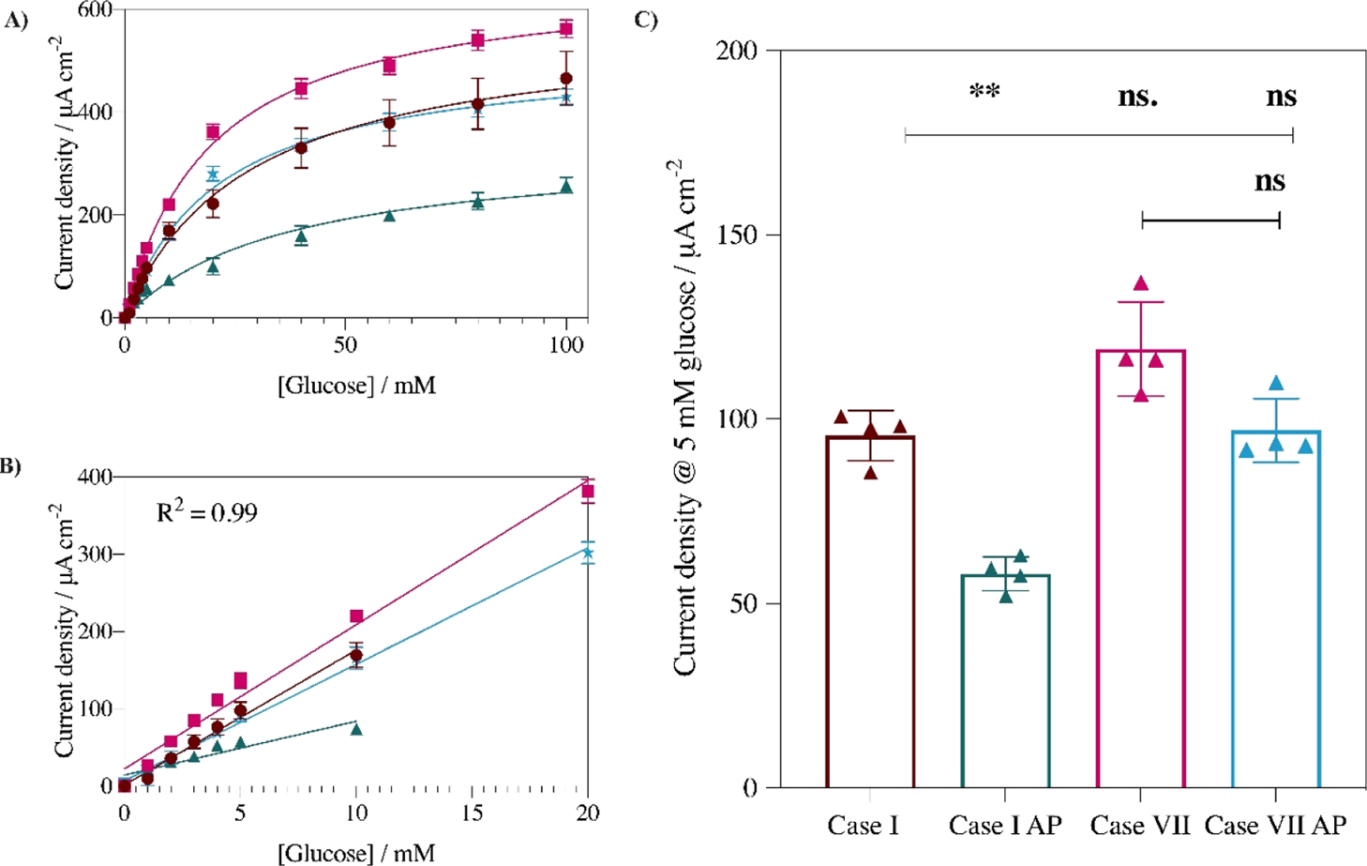
Glucose response for the amperometric biosensors tested at a constant applied potential of 0.35 V vs Ag/AgCl (3 M KCl) at 37 °C in PBS (0.05 M, pH 7.4) and artificial plasma (AP) presented as current density versus glucose concentration from (A) 0 to 100 mM and (B) response over the glucose concentration linear range (C) current densities of Case I and Case VII in 5 mM glucose and either PBS or artificial plasma (AP). Mean ± SD. (*n* = 4); *p < 0.03, **p < 0.002, ***p < 0.0002.

The response in 5 mM glucose of the novel polymer design Case VII sensor is compared to an uncoated control Case I sensor operating in PBS and artificial plasma (Figure 4). To prepare the novel polymer design Case VII sensors, both the negatively charged polymer P(VI^1^-SSNa^1^) as the interlayer and the MPC as the outer layer overcoats are applied in equal amounts (10 μg). The amperometric glucose oxidation current of the novel polymer design Case VII shows slightly higher current density (20%) than the uncoated control Case I sensor in PBS. This can be attributed to the high ionic conductivity of zwitterionic polymers, which enables rapid counterion movement. Additionally, the retention of high current densities means that glucose transport through the P(VI^1^-SSNa^1^) and MPC films encounters a low diffusional barrier. This behavior is similar to that reported previously, which is proposed to be due to chemical cross-linking between layers, leading to interlayer mixing.^14^ The novel polymer design Case VII multi-layer does not significantly affect the current density signal or the sensitivity of the biosensors in PBS, displaying a 12% improvement in sensitivity (Table 2) compared to the uncoated control Case I system. The novel polymer design Case VII system also performs better than a system containing Os(bpy)PVI and coated with 0.5 w/v % Nafion^44^ as the Nafion overcoat on those biosensors containing GOx and FAD-GDH resulted in 67 and 72% loss in sensor signal.

**Table 2:** Analytical Parameters for Glucose

sensor	*K*_M_^app^/mM	*j*_max_/μA cm^–2^	sensitivity/μA cm^–2^mM^–1^	LOD/mM
Case I in PBS	25.2 ± 4.7	557.1 ± 8.3	17.5 ± 1.9	2.9
Case I in artificial plasma	30.7 ± 3.8	315.2 ± 12.3	6.9 ± 1.3	5.3
Case VII in PBS	17.2 ± 5.1	520.5 ± 11.2	19.7 ± 0.9	1.7
Case VII in artificial plasma	23.1 ± 6.2	393.8 ± 30.2	15.1 ± 0.6	4.5

The advantage of using the polymer design system is highlighted by the differences between the novel polymer design Case VII and the uncoated control Case I sensor performance measured in artificial plasma. Some loss in the current densities is expected when switching to complex media; however, the loss is significantly less pronounced for the novel polymer design Case VII sensor with the multi-layer protective shield. The uncoated control Case I shows a 39% loss in current density in AP compared to that in PBS, whereas the novel polymer design Case VII shows a statistically insignificant loss of 9% under the same conditions (Figure 4C). The use of multi-layer coating also extends the range over which a correlation co-efficient of at least 0.99 is achieved for the linear fit of the current density response to concentration. This range is limited to 10 mM for the uncoated control Case I in PBS and AP but extends to 20 mM for the novel polymer design Case VII in PBS and AP (Figure 4B). Finally, the sensitivity data extracted from the slope of the linear range (Table 2) indicates that the novel polymer design Case VII sensor displays higher sensitivity than the uncoated control Case I, both in PBS and in artificial plasma. The uncoated control Case I sensor shows a 60% loss in sensitivity operating in artificial plasma compared to in PBS, whereas the novel polymer design Case VII sensor shows only a 23% loss in sensitivity.

It should be noted that the novel polymer design Case VII sensor shows similar sensitivity in artificial plasma to that of the uncoated control Case I sensor in PBS, despite being in complex media. The study reporting on the use of Nafion coating over Os(bpy)PVI-based enzyme electrodes^44^ does not provide data on sensitivity in artificial plasma, but the slopes from the linear portion of the Michaelis–Menten curves clearly indicate that operation in artificial plasma results in a significant loss in sensitivity. A study of sensors based on GO*x* encapsulated in poly(3,4-ethylenedioxythiophene) (PEDOT) or a system where PEDOT was functionalized with the zwitterionic polysulfobetaine (PSPEDOT) showed results similar to those observed in this study.^49^ Tests in PBS and plasma show that zwitterionic polymer systems provide higher current and sensitivity compared to non-zwitterionic counterparts, likely due to the influence of high ionic conductivity. Sensitivities for PSPEDOT and PEDOT of 12.63 and 7.54 μA cm^–2^ mM^–1^, respectively, in PBS decreased by 13 and 40% in plasma. While the introduction of a sulfobetaine zwitterionic moiety into the coating increased sensor sensitivity, similar to that observed here, the sensitivity for the novel polymer design Case VII sensor is higher than that reported for the PSPEDOT sensor.^49^ While many other studies target the application of glucose sensors in CGMs^50−52^ and include testing in complex media, the changes in analytical parameters and sensitivity in the complex media are not reported.

**Figure 5:**
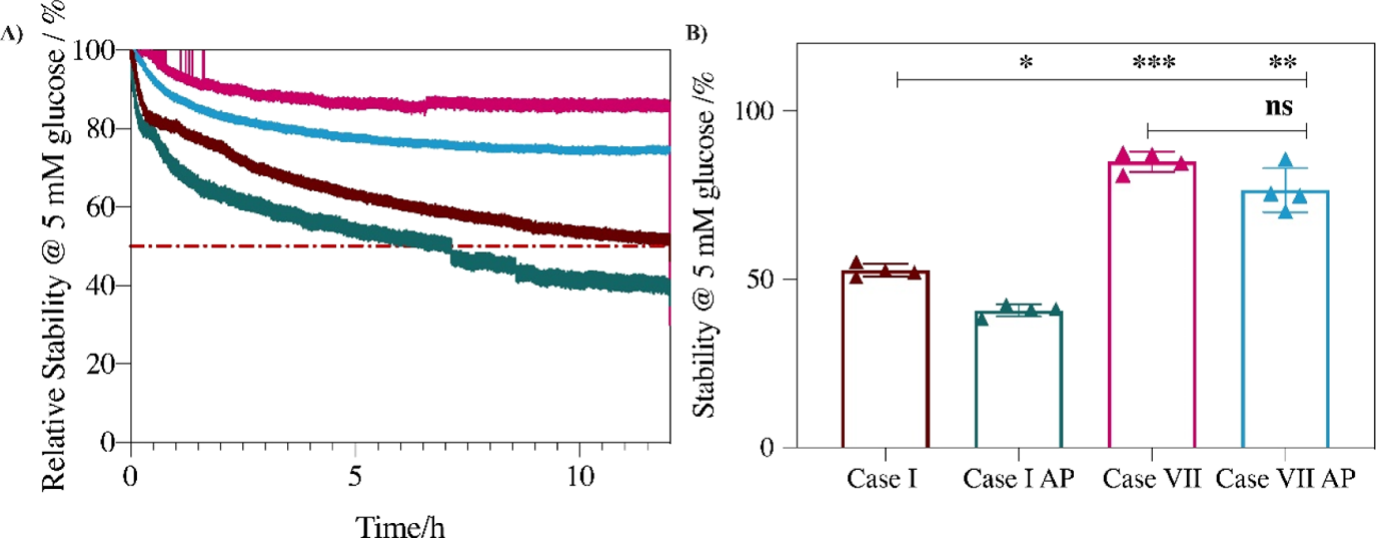
Operational stability over 12 h of amperometric response to 5 mM glucose for uncoated control Case I and novel polymer design Case VII systems in PBS (0.05 M, pH 7.4) and artificial plasma (AP) presented as (A) relative stability in sensor current density over time and (B) retained stability after 12 h for uncoated control Case I in PBS (maroon) and in AP (green) and for novel polymer design Case VII in PBS (pink) and in AP (blue). Mean ± SD. (*n* = 4); *p < 0.03, **p < 0.002, ***p < 0.0002.

The results on operational stability measured in PBS and artificial plasma and presented in Figure 5 confirm that multi-layer coating enhances the stability of the sensor. This is likely due to the stabilizing effect that polymer overcoats can have on the redox polymer sensing layer. The novel polymer design Case VII sensor shows better operational stability, with 85% of the signal retained after 12 h of continuous operation in PBS and 77% of the signal retained in artificial plasma over the same duration. This compares favorably to the uncoated control Case I response, which shows 54% stability in PBS and 38% in artificial plasma. Moreover, on comparing stability for the novel polymer design Case VII sensors to responses of sensors protected by individual polymer coatings (MPC-coated Case II and Nafion-coated Case III, Figure 2), it is evident that multi-layer coating provides improved protection over that provided by individual layers for sensors operating in artificial plasma. The use of a multi-layer coating in the novel polymer design Case VII compares well with the results of Nafion-coated GO*x*/MWCNT-based biosensor systems studied by Bennett et al.,^17^ despite the lack of use of a nanosupport in the novel polymer design Case VII system.

## Conclusions

Single-layer and multi-layer polymer coatings based on a range of protection mechanisms were investigated as anti-interference shields over a second-generation-mediated glucose biosensor. The sensor architectures were designed to target protection from either biological or anionic electroactive UA and AA interferences or from both types of interference. The study of the effect of the two most detrimental interferents, BSA and uric acid, showed that UA and biological interferents have individual effects on current density and operational stability that are compounded in complex media such as artificial plasma. For targeting protection against negatively charged low molecular-weight interferences, the effectiveness of negatively charged polymer layers for the electrostatic repulsion of the interferents was compared to a protective layer containing enzymes that consume the UA and AA interfering species. Due to the low activity of enzymes, the use of negatively charged polymer layers for the electrostatic repulsion of UA and AA interferences was more effective. An interference screening performed in complex media showed that the single-layer coatings examined in this study had at least one component acting as an interferent. Multi-purpose architectures where the outer layer consisted of an antifouling zwitterionic polymer (MPC) and an inner layer of either Nafion or a synthesized polyvinylimidazole-polysulfostyrene copolymer (P(VI^1^-SSNa^1^)) showed good resistance against interferences. The novel polymer design architecture consisting of P(VI^1^-SSNa^1^) and MPC was superior as it displayed low MARD values for all tested interferences, low variability in the obtained results, and a significantly better glucose sensor signal compared to the multi-layer system with the Nafion inner-layer. In contrast to other reports using polymer coatings, the application of the polymer design multi-layer did not negatively affect the current density or sensitivity of the biosensors. Additionally, the linear range of the sensor was wider for the multi-layer system than for the uncoated control. Overall, a multi-layer protective coating system consisting of the newly designed P(VI^1^-SSNa^1^) and MPC shows promise for application in biosensor development for CGMs.

## Supporting Information Available

Structures and NMR characterization of the negatively charged polymers, amperometry results in the presence of interferences, and sensor characteristics (PDF)
