# Supplementary material for: Cross-Linkable Polymer-Based Multi-layers for Protecting Electrochemical Glucose Biosensors against Uric Acid, Ascorbic Acid, and Biofouling Interferences
Source: ACS Sens. 2023 Mar 21;8(4):1756–65. doi: 10.1021/acssensors.3c00050 (PMC10152486; doi:10.1021/acssensors.3c00050)
Supplement: Supplementary file 1 — se3c00050_si_001.pdf [file se3c00050_si_001.pdf]

# Supplementary Information

**Cross-linkable polymer-based multi-layers for protecting electrochemical glucose biosensors against uric acid, ascorbic acid and biofouling interferences**

Anna Lielpetere[1†], Kavita Jayakumar[2†], Dónal Leech[2*] and Wolfgang Schuhmann[1*]

[1]Analytical Chemistry–Center for Electrochemical Sciences, Faculty of Chemistry and Biochemistry, Ruhr University Bochum, Universitätsstr. 150, 44780 Bochum, Germany
[2]School of Biological & Chemical Sciences, University of Galway, University Road, Galway, Ireland H91 TK33

[†]These authors contributed equally to the research

[*]Corresponding authors Wolfgang Schuhmann wolfgang.schuhmann@rub.de and Dónal Leech donal.leech@universityofgalway.ie

**Materials and methods**

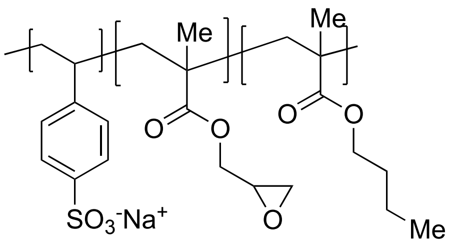


**P(SS-GMA-BMA)**

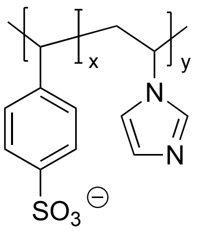


**P(VI[12]-SSNa[1]) / P(VI[1]-SSNa[1])**

Figure S1: Chemical structures for the negatively charged polymers tested. Monomer ratio in P(SS-GMA-BA) is SSNa:GMA:BA 5:3:2 while ratio in P($VI^{12}$-$SSNa^{1}$) is x = 12, y = 1 and ratio in P($VI^{1}$-$SSNa^{1}$) is x = 1, y = 1.

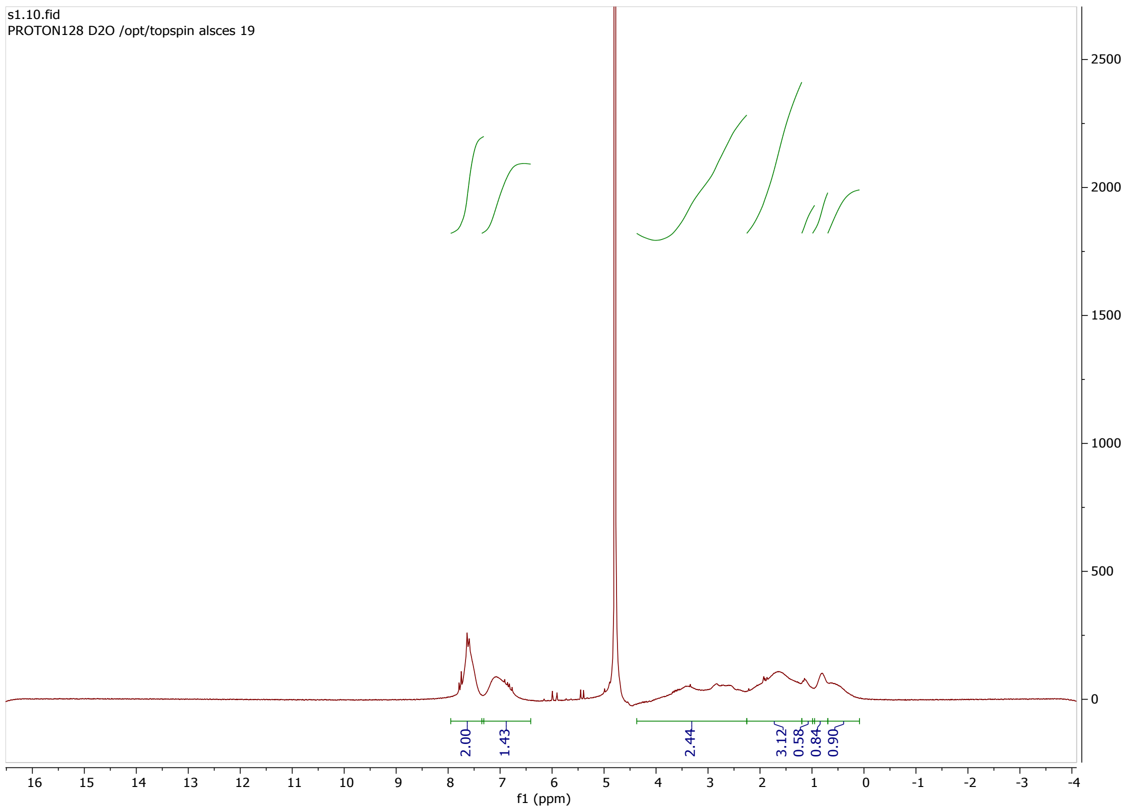


Figure S2. $^{1}H$-NMR (200 MHz, $D_2O$) spectrum of the P(SSNa-GMA-BA) polymer

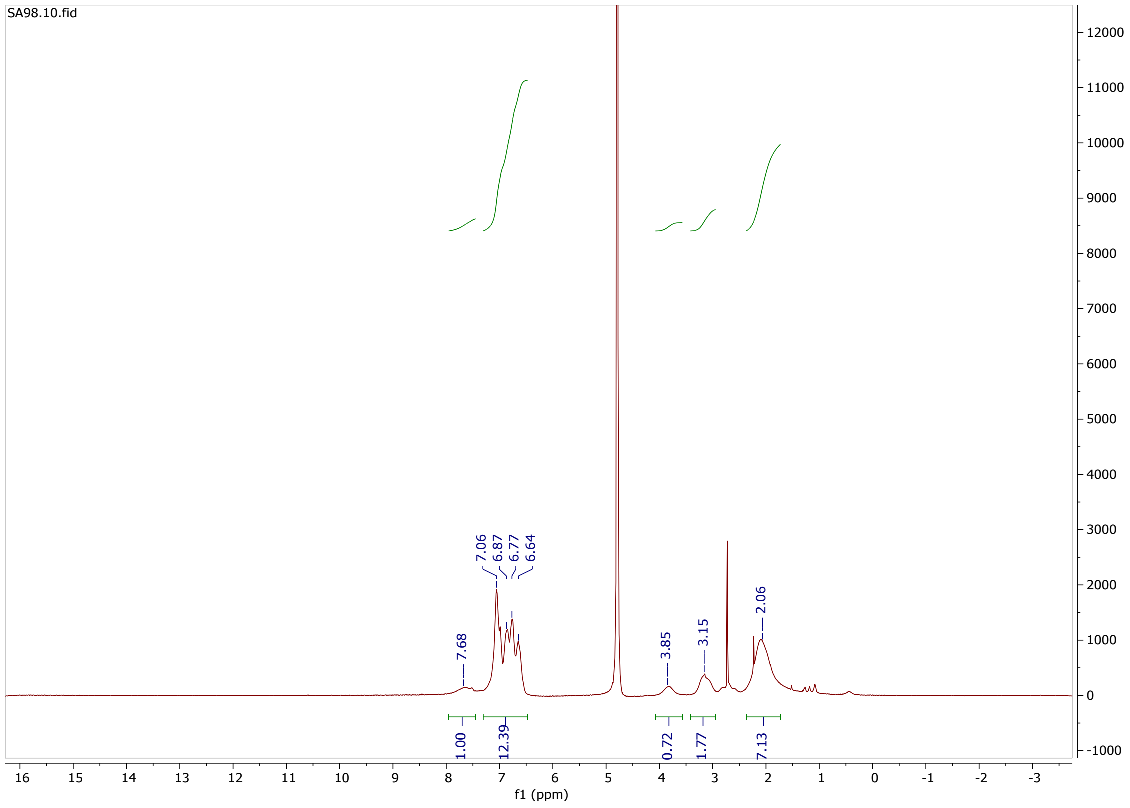


Figure S3. $^{1}H$-NMR (200 MHz, $D_2O$) spectrum of the P($SSNa^{1}$-$VI^{12}$) polymer

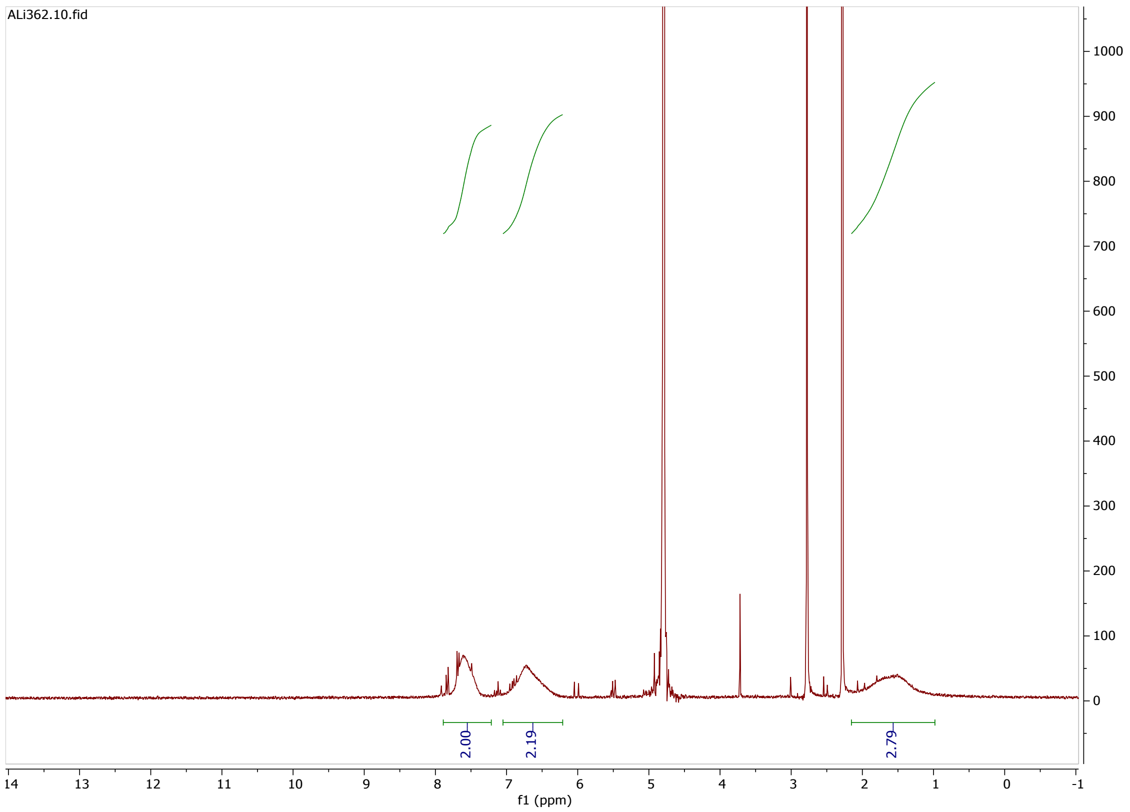


Figure S4. $^1$H-NMR (200 MHz, $D_2O$) spectrum of the P(SSNa$^1$-VI$^1$) polymer

**Supplementary results**

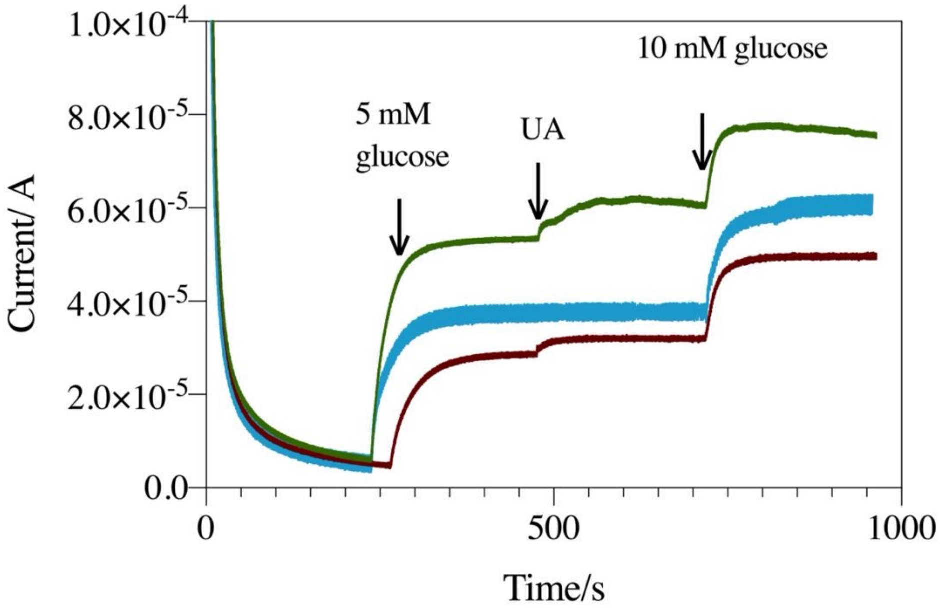

Figure S5: Amperometry at 350 mV for the system coated with P(SSNa$^{5}$-GMA$^{3}$-BA$^{2}$) (green), P(VI$^{12}$-SSNa$^{1}$) (red) and P(VI$^{1}$-SSNa$^{1}$) (blue) in PBS (0.05 M, pH 7.4).

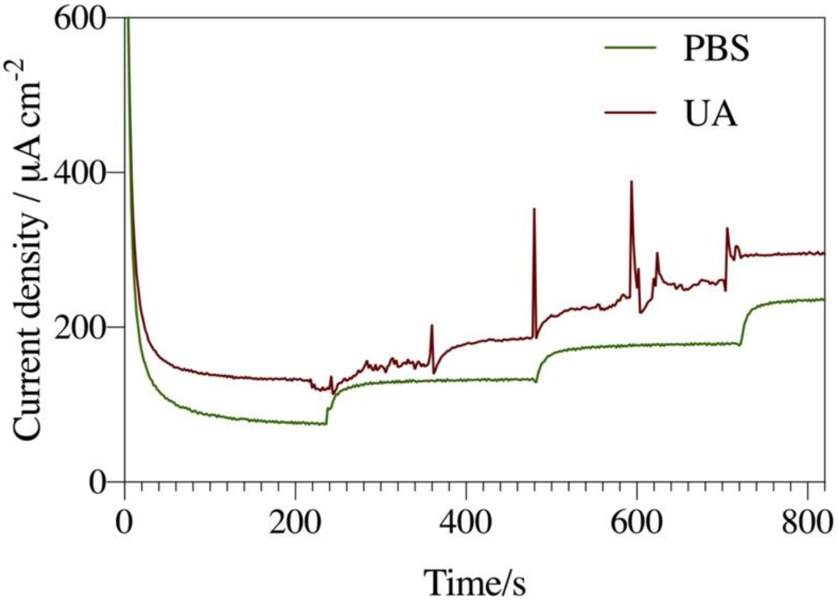


Figure S6: Amperometry at 350 mV for the uncoated control Case I in PBS (0.05 M, pH 7.4) and PBS containing uric acid at physiological concentration.

**Table S1.** $K_M^{app}$ and $j_{max}$ for uncoated control Case 1, and systems coated with MPC Case II and Nafion Case III in PBS and artificial plasma. (n = 3, Mean ± SD)

| **Media** | **Non-coated control** | | **MPC** | | **Nafion** | |
|---|---|---|---|---|---|---|
| | **$K_M^{app}$ / mM** | **$j_{max}$ / µA cm$^{-2}$** | **$K_M^{app}$ / mM** | **$j_{max}$ / µA cm$^{-2}$** | **$K_M^{app}$ / mM** | **$j_{max}$ / µA cm$^{-2}$** |
| PBS | 26.2 ± 1.1 | 557 ± 11 | 13.7 ± 3.1 | 455 ± 13 | 23.4 ± 4.7 | 261 ± 13 |
| Artificial Plasma | 23.0 ± 1.4 | 394 ± 8 | 15.5 ± 0.1 | 427 ± 13 | 81 ± 10 | 218 ± 11 |

| | | | | | | |
|---|---|---|---|---|---|---|
| BSA | 7.3 ± 1.2 | 292 ± 8 | 14.6 ± 4.8 | 486 ± 20 | 69.8 ± 5.1 | 278 ± 8 |
| Uric Acid | 14.3 ± 4.5 | 285 ± 14 | 10.9 ± 1.2 | 249 ± 2 | 20.1 ± 3.2 | 296 ± 11 |

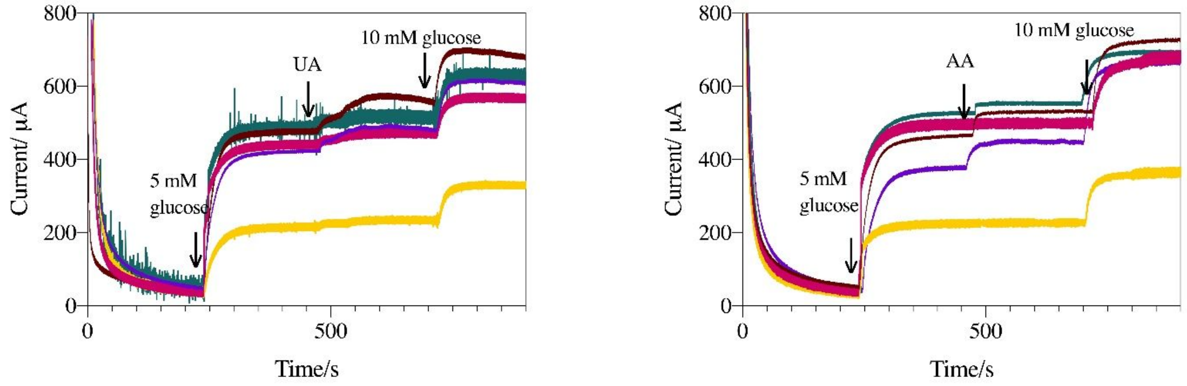


Figure S7: Amperometry at 350 mV for the uncoated control Case I (dark red), MPC Case II (green), P(VI[1]-SSNa[1]) Case IV (yellow), enzyme layer Case V (violet) and novel polymer design Case VII (pink) in PBS (0.05 M, pH 7.4) with injections of A) uric acid and B) ascorbic acid.

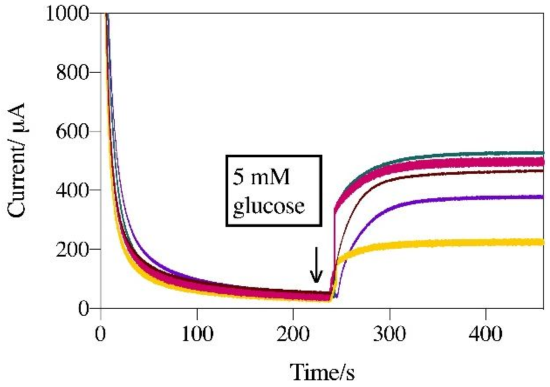


Figure S8: Expanded view of figure S7 amperometric response at 350 mV for the uncoated control Case I (dark red), MPC Case II (green), P(VI[1]-SSNa[1]) Case IV

(yellow), enzyme layer Case V (violet) and novel polymer design Case VII (pink) in PBS (0.05 M, pH 7.4) with injections 5 mM glucose.
